# Supplementary material for: MetaQTL: a package of new computational methods for the meta-analysis of QTL mapping experiments
Source: BMC Bioinformatics. 2007 Feb 8;8:49. doi: 10.1186/1471-2105-8-49 (PMC1808479; doi:10.1186/1471-2105-8-49)
Supplement: Additional File 7 — MetaQTL Package : jar file and tutorial. This Zip archive contains both the MetaQTL JAR file and the files of the tutorial. [file 1471-2105-8-49-S7.zip › org.inra.metaqtl/doc/index-files/index-6.html]

F-Index


|  |  |  |
| --- | --- | --- |
| **Overview** \| Package \| Class \| Use \| **Tree** \| **Deprecated** \| **Index** \| **Help** | |  |
| **PREV LETTER**   **NEXT LETTER** | **FRAMES**    **NO FRAMES**     **All Classes** |  |


A B C D E F G H I K L M N O P Q R S T U V W X Y Z 

---


## **F**

**F** - Variable in class org.metaqtl.util.OffspringManager.HaldaneWaddingtonProportion: **F\_coeff** - Static variable in class org.metaqtl.util.OffspringManager.HaldaneWaddingtonProportion: **fisherVariance(double, int, int, int)** - Static method in class org.metaqtl.util.OffspringManager: Returns the inverse of the Fisher Information depending on the type of cross for a given recombination rate and a given population size. **fixCluster()** - Method in class org.metaqtl.ChromCluster: This methods frozes the index of the distinct markers over all the members of the cluster. **font2string(Font)** - Static method in class org.metaqtl.graph.MetaGraphPar: **formatDouble(double)** - Static method in class org.thalia.bio.util.NumberFormat: **frames** - Variable in class org.metaqtl.CMarkerSequence: The frame of the common marker sequences **from** - Variable in class org.metaqtl.Qtl.QTLSupport: The position from the first marker to the left border of the IC. **fromEntity(IBioEntity, BioEntityBean)** - Static method in class org.thalia.bio.entity.adapter.BioEntityAdapter: **fromEntity(IBioEntity)** - Method in class org.thalia.bio.entity.adapter.BioEntityCollectionAdapter: **fromEntity(IBioEntity)** - Method in class org.thalia.bio.entity.adapter.GeneticMapBeanAdapter: **fromEntity(IBioEntity)** - Method in class org.thalia.bio.entity.adapter.LGroupBeanAdapter: **fromEntity(IBioEntity)** - Method in class org.thalia.bio.entity.adapter.LocusBeanAdapter: **fromEntity(IBioEntity)** - Method in class org.thalia.bio.entity.adapter.OntologyBeanAdapter: **fromEntity(IBioEntity)** - Method in class org.thalia.bio.entity.adapter.OntologyTermBeanAdapter: **fromEntity(IBioEntity)** - Method in interface org.thalia.bio.IBioAdapter: Convert the given compliant object to a bio entity object. **fromQTL(Qtl)** - Static method in class org.metaqtl.adapter.QtlAdapter: This method converts a `Qtl` into an object which class implements the `IBioLocus interface. func(double) - Method in interface org.metaqtl.numrec.NumericalUtilities.Function: Returns the value of the function in x. funcd(double) - Method in interface org.metaqtl.numrec.NumericalUtilities.NRFunction: Compute both the value and the derivate of the function in x. function - Variable in class org.thalia.bio.entity.bean.OntologyBean: The function of the ontology.`

---


|  |  |  |
| --- | --- | --- |
| **Overview** \| Package \| Class \| Use \| **Tree** \| **Deprecated** \| **Index** \| **Help** | |  |
| **PREV LETTER**   **NEXT LETTER** | **FRAMES**    **NO FRAMES**     **All Classes** |  |


A B C D E F G H I K L M N O P Q R S T U V W X Y Z 

---
